# Health Behaviors in the LGB+ Population: Variation Across Adulthood

**DOI:** 10.1093/geroni/igab046.3074

**Published:** 2021-12-17

**Authors:** Jessica Noblitt, Anne Barrett

**Affiliations:** Florida State University, Tallahassee, Florida, United States

## Abstract

Health behaviors, which predict physical and mental health, are patterned by social factors, with some groups engaging in more health-enhancing behaviors than others. LGB+ people face more economic and social barriers to participation in healthy behaviors, along with the stress of discrimination that could lead to unhealthy behaviors to cope. Although some studies have examined variation in health behaviors by sexual identity, they focus almost exclusively on adolescents and young adults. However, such differences may decline across adulthood, as stress related to sexual identity declines with age among LGB+ individuals. Addressing this issue, we use data from the National Health Interview Survey (2016-2018) to examine differences by sexual identity in substance use, weight-related behaviors, healthcare utilization, and sleep. We compare the patterns across three age groups – young, middle-aged, and older adults. Results for each health behavior reveal that differences by sexual identity are indeed greatest among young adults. The magnitude is smaller in middle age, and no significant differences by sexual minority status are observed at older ages.

